# Willingness to share personal health record data for care improvement and public health: a survey of experienced personal health record users

**DOI:** 10.1186/1472-6947-12-39

**Published:** 2012-05-22

**Authors:** Elissa R Weitzman, Skyler Kelemen, Liljana Kaci, Kenneth D Mandl

**Affiliations:** 1 Children’s Hospital Informatics Program, Children’s Hospital Boston, Boston, MA, USA; 2 Division of Adolescent Medicine, Children’s Hospital Boston, Boston, MA, USA; 3 Division of Emergency Medicine, Children’s Hospital Boston, Boston, MA, USA; 4 Department of Pediatrics, Harvard Medical School, Boston, MA, USA; 5 Manton Center for Orphan Disease Research, Children’s Hospital Boston, Boston, MA, USA

## Abstract

**Background:**

Data stored in personally controlled health records (PCHRs) may hold value for clinicians and public health entities, if patients and their families will share them. We sought to characterize consumer willingness and *un*willingness (reticence) to share PCHR data across health topics, and with different stakeholders, to advance understanding of this issue.

**Methods:**

Cross-sectional 2009 Web survey of repeat PCHR users who were patients over 18 years old or parents of patients, to assess willingness to share their PCHR data with an-out-of-hospital provider to support care, and the state/local public health authority to support monitoring; the odds of reticence to share PCHR information about ten exemplary health topics were estimated using a repeated measures approach.

**Results:**

Of 261 respondents (56% response rate), more reported they would share all information with the state/local public health authority (63.3%) than with an out-of-hospital provider (54.1%) (OR 1.5, 95% CI 1.1, 1.9; p = .005); few would not share any information with these parties (respectively, 7.9% and 5.2%). For public health sharing, reticence was higher for most topics compared to contagious illness (ORs 4.9 to 1.4, all p-values < .05), and reflected concern about anonymity (47.2%), government insensitivity (41.5%), discrimination (24%). For provider sharing, reticence was higher for all topics compared to contagious illness (ORs 6.3 to 1.5, all p-values < .05), and reflected concern for relevance (52%), disclosure to insurance (47.6%) and/or family (20.5%).

**Conclusions:**

Pediatric patients and their families are often willing to share electronic health information to support health improvement, but remain cautious. Robust trust models for PCHR sharing are needed.

## Background

Clinicians and public health authorities need access to accurate and complete health data in order to make timely and appropriate health decisions. Medical records, a vital source of health data, are fragmented and often lack vital information about health behaviors, treatment adherence and side effects [1,2]. When comprised of aggregated data from medical records, public health data share these weaknesses [3]. Problems are amplified in pediatrics: children’s experiences of their disease state and quality of life may be different from perceptions of external observers [4,5]. Most health record systems don’t support capture of child or parent reports, constraining the acuity of health record data and resultant quality of care [1,6-8]. While bringing improved data to the point-of-care and to public health decision-makers is at the heart of our national investment in health information technology and care improvement efforts [9,10], few proven cost-efficient approaches to doing so exist.

One novel approach to bringing improved data into clinical care and public health may be the *personally controlled health record* (PCHR)—an individually controlled Web-based platform that integrates personally reported as well as clinically and administratively sourced data over sites of care and time [11-13]. The model is a digital web-based collection of a patient’s medical history in which copies of medical records, reports about diagnosed medical conditions, medications, vital signs, immunizations, laboratory results, and personal characteristics like age and weight can be stored. Designed to integrate medical and social information across sites of care and over time, the pchr is readily accessible to and controlled by *individuals*[14]. As such, it may generate high levels of patient buy-in, use and long-term commitment including for health monitoring and research. As a web-based communications and service *platform*, the PCHR might serve as a lifelong record to promote patient engagement and activation in disease management, communication with clinicians, and shared decision-making. Aggregations of data stored in PCHRs may provide a vital new source of public health information if patients consent to sharing their data. Consented sharing of personal health information (PHI) stored in a PCHR could augment availability of actionable health data without burdening clinicians or impacting workflows [15]. Patients could authorize data sharing from their PCHR with collaborating clinicians to foster care coordination and/or with public health authorities to support surveillance and response. While this seems logical and efficient, the model relies on patient willingness to share data and, for public health especially, “information altruism” [16].

Our studies with early adopters and testers of PCHRs suggest that adults are willing to share their PCHR data for public health research or non-commercial use under conditions of consent and anonymity [12,17]—conditions that also support sharing of traditional medical records for research [18-20]. Early results with adults beg further study in the pediatric setting; are pediatric patients and/or their parents/guardians willing to share PCHR data? Are they willing to share different kinds of data with different decision makers? Are they willing to share data comprehensively or are they reticent to share select information? Concern about disclosure, discrimination and stigma have impeded sharing of traditional medical records [21,22] and may impede PCHR sharing too. Reticence may be especially high for social, behavioral and mental health data commonly construed as sensitive [23-25] despite their relevance to understanding and improving service use, treatment efficacy and outcomes [26-30].

We investigated parent and young adult attitudes toward sharing PHI stored in a pediatric PCHR with clinical and public health decision-makers. We hypothesized high willingness to share most information and reticence to share sensitive information, anticipating no differences in willingness to share across clinical and public health targets. Findings are anticipated to be of interest to clinicians whose understanding of their patients’ conditions and resultant clinical guidance and decision-making may be impacted by access to data patients share. Understanding tendencies of patients to share PHI selectively through the PCHR might lead clinicians to ask patients more targeted questions about potentially sensitive health issues, elucidating higher quality information. Public health decision makers might use aggregated PCHR data shared by patients to inform surveillance activities, needs assessments, and planning and evaluation. As with all efforts relying on citizen-reported data, including for example the Behavioral Risk Factor Surveillance System [31] and the National Health and Nutrition Examination Survey [32] there are important biases in response.

## Methods

We conducted a cross-sectional Web-based survey of attitudes and practices germane to sharing health information from a PCHR, to support patient care and public health monitoring. All participants provided informed consent using an online form prior to taking the survey. The Children’s Hospital Boston Institutional Review Board reviewed and approved all study activities.

### Target sample and eligibility criteria

We targeted patients and/or parents/guardians of patients who were repeat users of the open source indivo PCHR [33], embedded in a hospital patient portal system. We defined repeat PCHR users as those who had logged in to the system on at least five separate occasions, as recorded in system tracking data. Respondents were at least 18 years old and could read English.

### PCHR technology model

The PCHR system with which respondents were familiar contained clinically and administratively entered data about diagnoses, vital statistics, allergies, medications, lab reports, problems and health behaviors populated from the electronic medical record at the host institution. Respondents could review, annotate but not delete from their record and set role-based access permissions for others to see it, including out-of-hospital providers, however sharing with public health authorities was not an option. A secure messaging and an online scheduling system operate with the PCHR and a policy framework governs access and sharing rules in the pediatric domain [34]. An online interactive tutorial and demonstration system provides audio-narrated instruction to orient users to the PHCR and to its functions including reviewing, annotating and communicating with clinicians.

### Study domains and measures

The survey primarily collected closed-ended, fixed-choice data. Some questions had open-ended fields for narrative comment. Domains included perceived value of the PCHR and its capabilities, knowledge and history of sharing information from the PCHR, willingness to share data from the PCHR, conditions and contexts for sharing, demographics and health status. *Un*willingness to share specific categories of health information, or reticence, was assessed with the question “Are there any categories of information included in your/your child's PCHR that you would consider sensitive and would not share?” asked separately for sharing with: 1) an out-of-hospital health care provide (“outside provider”); and 2) the state/local public health department. Respondents could indicate which items from a list in the survey were too sensitive to share (multiple selections allowed). The list included: contagious illness, violence, sexually transmitted diseases (STD), tobacco, alcohol, other substances, genetic disorders, mental illness, family information and financial information. Participants were classified as willing to share *all* categories of information with a target if they either chose “I would share all categories of information” as a response *or* if they did not check off any information category as too sensitive to share. The context for sharing with an outside provider was to support patient care, and the context for sharing with public health was to support health monitoring and research.

Perceived values of the PCHR and reasons for reticence to share were based upon measures described previously [17,35].

Demographic and self-rated health descriptors were based on standardized measures. Race and ethnicity reports were reduced for analyses to white/non-white categorizations. Respondents were classified as non-white unless they reported solely Caucasian race and also non-Hispanic ethnicity. Patient self- (or parent)-rated health was based on a scale question where values of “good”, “very good” or “excellent” were used to indicate a better health condition and compared to values of “fair” or “poor”. Patient age in years was reported and dichotomized at 13 years and older for categorical analyses. Categorical responses for household income were reported for the values of under $40,000, $40,000-100,000, $101,000-200,000, and over $200,000 and dichotomized at over $100,000 for analyses.

To measure response bias, we compared patient characteristics of responders to the source population of all PCHR patient records that existed at the time of survey (n = 1610 inclusive of responders), obtained from an administrative database. We compared aggregate data about the source population to those for responders for measures of patient: age, race/ethnicity, duration of treatment at the hospital, and in-state residence.

### Response rate

An analysis of system data identified 820 repeat users of the system all of whom were notified by email that a bulletin communication had been posted to their PCHR homepage. To protect patient privacy, this email made no mention of the nature of the bulletin or presence of a survey. Of the notified users, 463 (56.5%) were active in the portal system during the study period, as evident by information about last login, and could reasonably be assumed to have had an opportunity to see the survey notification bulletin. Of these active users, 261 took the survey for a response rate of 56%. Responders did not differ from the source population in measures of patient race, length of care at the hospital, or in-state residence. Patients in the source population were more likely to be over the age of 12 (40% versus 31%, p = .006).

### Analyses

Descriptive statistics characterized response rate and sample demographics. Observations in which respondents selected a “prefer not to answer” option were excluded from analyses except for analyses of income and sharing where missing income data were substantial and responses separately categorized. Narrative responses were reviewed and when appropriate, assigned a coded value by matching responses to *a priori* fixed response fields, or preserved as written for qualitative annotation. Chi Square tests compared respondents and the source population and assessed associations between willingness to share by topic and demographics. Willingness to share was measured by topic as a dichotomous dependent variable. We used a repeated measures analytic approach to compare sharing probabilities for respondents with respect to different topics and different “targets” defined as outside providers and public health, recognizing that responses to sharing questions are clustered at the individual level. All analyses were performed using SAS 9.2.

## Results

### Sample characteristics

Of the 261 respondents, 21 (8%) were patients and the rest were parents or guardians of patients. Demographic and health characteristics of the survey participants are described in Table 1.

**Table 1:** Characteristics of the sample

Total N = 261	N (%)
Patient age 0–12 years	178 (68.2)
2+ children in household	141 (61.8)
White, non-Hispanic	209 (91.3)
Income over $100,000	67 (44.1)
Does not report income	74 (32.5)
Fair or poor self-rated health	40 (17.5)
Received care at site < 2 years	48 (21.2)

### Interest in maintaining the PCHR and understanding of sharing

Among survey respondents, interest in the PCHR was nearly universal with 97.1% indicating that they would like to maintain their record as they/their child matures. Knowledge about sharing through the PCHR was limited; 42.3% were aware that they could allow other individuals to view their record. Only 22.6% of the total sample knew that they could choose to share only portions of their record. Only 13.8% (n = 33) of all respondents indicated that they had previously shared their PCHR. Among these, the most common target for sharing was with an outside provider, reported by 66%. Those who reported having shared their record were no more likely to report knowledge of PCHR sharing capabilities than those with no history of sharing (16 reported knowledge of sharing; OR 1.3, 95% CI 0.6, 2.8, p = .4). A history of sharing the PCHR was not associated with willingness to share PCHR information about different topics with public health or providers.

### Perceptions of PCHR value

**Figure 1:**
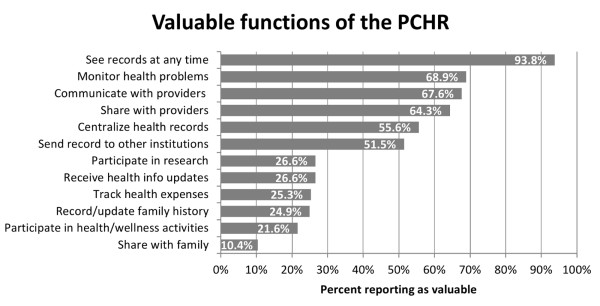
Percent of participants reporting they value specific functions of the PCHR.

Patient perceptions about the value of the PCHR focused mainly on personal control over and access to health records (Figure 1). Large percentages of respondents also reported valuing the PCHR as a tool for interacting with or sharing records with other providers or institutions. When asked to indicate which single function was most important of the many they valued, 64.7% chose unlimited access to their record, followed by centralizing health records (9.1%). Value selections were not associated with child age, health status, length of treatment at the hospital, history of sharing, or willingness to share information with public health or an outside provider. No clear pattern was evident in associations among specific value areas and other factors, including number of children in a household, race or income. Interest in keeping the record was not uniformly associated with perceived value areas.

### **Reticence to share by target and health topic**

More respondents were willing to share all categories of information with the state/local public health authority (63.3%) than with an outside provider (54.1%) (OR 1.5, 95% CI 1.1, 1.9; p = .005). Few respondents reported they would not share *any* category of information with an outside provider or public health authority, respectively 5.2% and 7.9%.

**Figure 2:**
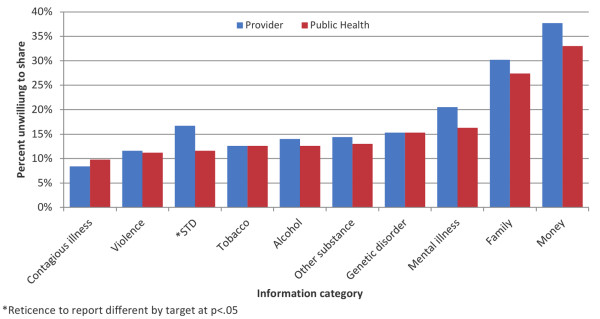
Reticence to share PCHR information by topic, and target.

Reticence to share PCHR information varied by health topic and was lowest with respect to sharing contagious illness information; only 8.7% and 9.2% of respondents were unwilling to share this information with a provider outside their home institution, or with the state/local public health authority, respectively (Figure 2). Reticence was highest with respect to sharing financial information; 37.6% and 32.3% of respondents were unwilling to share this information with an outside provider and a public health authority, respectively. Subjects were more likely to be reticent to share information on sexually transmitted disease with an outside provider than with public health (OR 1.6, 95% CI 1.03, 2.5, p = .037). No other differences in sharing across targets by topic were found.

### Reticence to share with an outside provider, by topic

**Figure 3:**
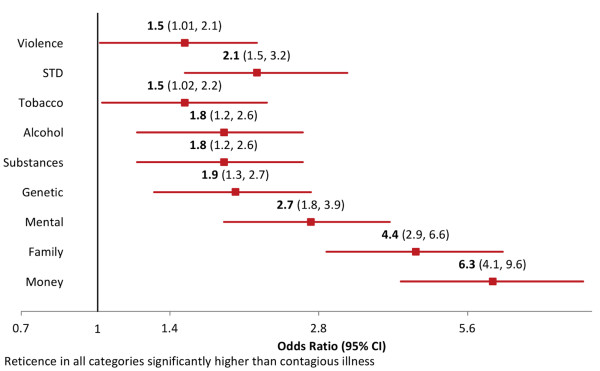
Across-topic odds participants are reticent to share different kinds of PCHR information with an outside provider, using contagious illness as the reference category.

**Figure 4:**
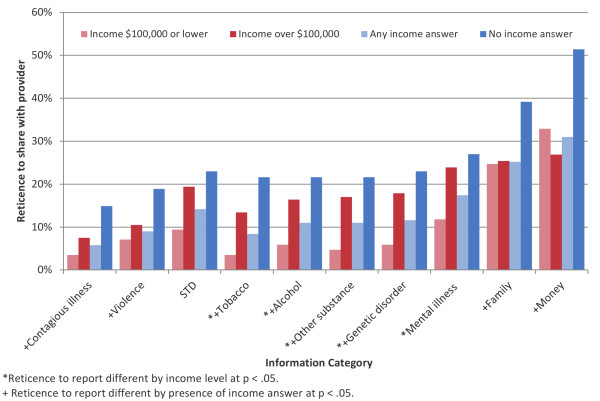
Reticence to share different kinds of PCHR information with an outside provider, stratified by reported income.

When asked to characterize willingness to share PCHR information with an outside provider, reticence was higher for all exemplary health topics compared to contagious illness (Figure 3). For example, respondents were 6.3 times more likely to report reticence to share information about money than contagious illness with an outside provider (p < .0001). For all topics assessed, willingness to share information with an outside provider was not associated with patient’s age, health status, number of children in household, or race. Reporting an income of $100,000 or lower, and reporting an income at all (versus not providing information about one’s income) were both positively associated with willingness to share health information for multiple health topics (Figure 4).

**Figure 5:**
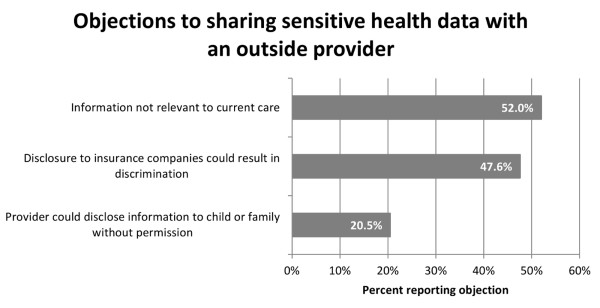
Percent of participants reporting specific reasons they are reticent to share sensitive health data with an outside provider for care improvement.

The most common objections to sharing data with an outside provider were relevance to patient care and the potential for discrimination by insurance companies (Figure 5). Narrative responses indicated that a decision about sharing categories of information would be made on a case-by-case basis, depending on the reason for consulting an outside provider, the patient/family relationship with the provider, and consideration of why information was needed or requested. A few parents noted that they would not share any information that might prejudice an outside provider’s judgment in diagnosing or treating their child.

### Reticence to share with public health, by topic

**Figure 6:**
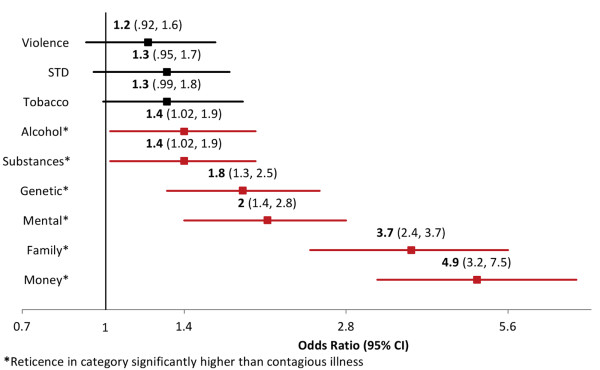
Across-topic odds participants are reticent to share different kinds of PCHR information with a public health authority, using contagious illness as the reference category.

**Figure 7:**
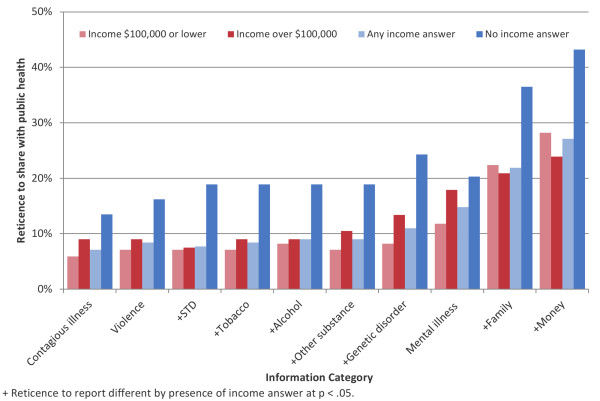
Reticence to share different kinds of PCHR information with a public health authority, stratified by reported income.

Compared to reticence about sharing contagious illness information with a public health authority, respondents were significantly more likely to report reticence to share information about all topics except violence, sexually transmitted diseases, and tobacco (Figure 6). For example, respondents were 4.9 times more likely to report reticence to share information about money compared to contagious illness with a public health authority (p < .0001). Willingness to share health data by category was not associated with patient’s age, race or health status, the number of children in the household, or income. Those who declined to provide their family income were less likely to share information from every category but mental illness, contagious illness and violence (Figure 7). There was no association between reported income level and willingness to share with public health in any information category.

**Figure 8:**
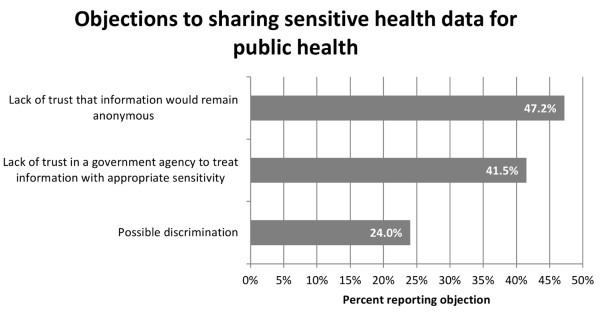
Percent of participants reporting specific reasons they are reticent to share sensitive health data with a public health authority.

Lack of trust in both the anonymity of data and in a government agency handling the data were the primary concerns reported for sharing with public health (Figure 8). In narrative comments, participants questioned the relevance of providing health data to a public health authority. Several respondents also commented that even with anonymized data, inclusion of personal information in a small sample could jeopardize privacy.

## Discussion

Among experienced users of a live pediatric PCHR we found moderate levels of willingness to share stored data with out-of-hospital providers and low levels of absolute reticence. We found moderately high levels of willingness to share PCHR data with public health authorities and low levels of absolute reticence. Overall, respondents were half again more likely to report they would share all of their PCHR data with a public health authority than with other providers. More than half of our sample was willing to share PHI stored in a PCHR with an out-of-hospital provider. We did not ask patients to share physician notes. Walker et al., found higher reticence for this for patients using a portal [36].

While a majority of respondents reported they would share information about every health topic queried, reticence varied by topic. For both sharing targets areas of least reticence concerned contagious illness, which may reflect norms conducive to sharing under mandatory reporting rules and/or goals of treatment and containment among social contacts [37,38]. For both sharing targets, areas of peak reticence pertained to money, family issues, and mental health. Odds for reticence in these areas were very high as hypothesized, and markedly high as well for information about alcohol and substance use, genetic information and STDs. While present for both targets, reticence affected a greater number of topics for sharing with providers.

Quantitative and qualitative reports suggest that reticence to share with both stakeholders reflects distrust in how data might be used, concern about disclosure and attendant risks for stigma and discrimination, lack of transparency and, for public health sharing only, concern for the preservation of anonymity and ability of a government agency to treat shared data with appropriate sensitivity. Stigma and discrimination are well-documented [39-43] making reticence a self-protecting impulse for some respondents. However, reticence was expressed for sharing data about social and behavioral health problems that are prevalent, poorly screened, and related to service use and adherence generally [44-47]. Thus, reticence may handicap decision making by clinical and public health authorities whose actions are guided by patient-reported and/or shared data, and could undermine use of the PCHR as a virtual medical home that serves as a bridge for collaborating clinicians [48].

Reticence varied by reported income for sharing with providers only, in which case lower income respondents expressed less reticence than higher income respondents. This is a provocative finding that could reflect myriad factors, including possibly lower expectations about one’s ability to control personal health information among lower income respondents as well as higher expectations about one’s ability to obtain private, personally-mediated care for sensitive or stigmatizing issues among higher income respondents. Income gradients are under-explored with regard to data sharing, and have not been found with respect to use of personal health record systems in general [49].

Reticence to share, especially with outside providers, seems inconsistent with perceived value areas of the PCHR—namely, to increase access to and centralization of health data, and to foster communication among providers. The perceived value of the PCHR in these areas is offset by concerns for clinical relevance, trust, discrimination and unauthorized disclosure of data. Findings underscore the need for transparent and trustworthy sharing mechanisms and comprehensive patient education on the risks and benefits of sharing PCHR information.

### Limitations

This is a single site study—findings may not generalize to other populations. We assessed attitudes across several but not all health areas, for two main but not all possible targets. A majority of eligible respondents participated. Demographic data about patients were available for the source population of PCHR users in aggregate. Comparison of aggregate sample characteristics for respondents and the source population identified no differences in patient race, length of treatment at the hospital or in-state geographic residence but found that patients in the source population were older. Available data do not afford direct comparison of responders and non-responders. Survey research has inherent limitations around capturing nuance and this may be important to understanding sharing of information from a still novel technology. Analyses of narrative data and reports about reasons for reticence are included to deepen our understanding of sharing. Alternative approaches were not used but might include surveying respondents about sharing under specific patient scenarios or focus group research.

## Conclusions

A majority of experienced PCHR users, most of them parents of pediatric patients, report they are willing to share personal health information with other providers to support patient care, and with public health authorities to support health management and research, across a range of health topics areas. Moreover, a relatively small percentage of users report absolute reticence. On the other hand, even among this sample of experienced and committed PCHR users, reticence levels are substantial around topics important to understanding patterns of service use, adherence and outcomes. Concerns about sharing reported by patients and parents/guardians are a wakeup call to engineer robust and trustworthy PCHR systems, to ameliorate concerns and support sharing. In parallel, efforts are needed to engage consumers in a conversation about the importance of sharing information in a comprehensive fashion to afford better understanding of a given patient and population.

## Abbreviations

HIT: Health information technology; PCHR: Personally controlled health record; PHI: Personal health information.

## Competing interests

The authors declare that they have no competing interests.

## Authors’ contributions

ERW conceptualized the study, designed the survey instrument, directed data analyses, interpreted study results, and had principal responsibility for drafting the manuscript. SK analyzed the study data and assisted in drafting the manuscript. LK helped design the survey instrument, managed data collection, and reviewed the final manuscript. KDM developed the core technology, reviewed the study design and data, helped interpret results and helped prepare the manuscript. All authors read and approved the final manuscript.

## Pre-publication history

The pre-publication history for this paper can be accessed here:

http://www.biomedcentral.com/1472-6947/12/39/prepub
